# Multimodal Spatial Calibration for Accurately Registering EEG Sensor Positions

**DOI:** 10.1155/2014/826019

**Published:** 2014-04-03

**Authors:** Jianhua Zhang, Jian Chen, Shengyong Chen, Gang Xiao, Xiaoli Li

**Affiliations:** ^1^College of Computer Science and Technology, Zhejiang University of Technology, Hangzhou 310023, China; ^2^National Key Laboratory of Cognitive Neuroscience and Learning, Beijing Normal University, Beijing 100875, China

## Abstract

This paper proposes a fast and accurate calibration method to calibrate multiple multimodal sensors using a novel photogrammetry system for fast localization of EEG sensors. The EEG sensors are placed on human head and multimodal sensors are installed around the head to simultaneously obtain all EEG sensor positions. A multiple views' calibration process is implemented to obtain the transformations of multiple views. We first develop an efficient local repair algorithm to improve the depth map, and then a special calibration body is designed. Based on them, accurate and robust calibration results can be achieved. We evaluate the proposed method by corners of a chessboard calibration plate. Experimental results demonstrate that the proposed method can achieve good performance, which can be further applied to EEG source localization applications on human brain.

## 1. Introduction

Photogrammetry is a measurement way to determine the object's position, shape, and size by collecting the image data in the three-dimensional space at a particular given moment in time. Photogrammetry achieves good achievements in the study of the localization problem of EEG sensors. Previous studies were based on CCD camera using the following three approaches to calculate the position of EEG sensors: (i) multiple cameras (capturing images data in multiple views), (ii) two cameras (stereophotogrammetry), and (iii) a single camera (either the camera is moving along a given path while the object is stationary or the camera is fixed and the object is moving).

Up to now, it is found that there are four photogrammetric methods, which are used in EEG system, presented in the literature. Bauer et al. [1] use 12 cameras to capture the electrodes, and it is concluded that their method is practical. Nevertheless different characteristics of the cameras might result in errors of localization. Russell et al. [2] propose another photogrammetric method that must be used with the associated product, the Geodesic Sensor Net, which increases the complexity in both system design and operation. In Baysal and Şengül's [3] system, a rotating digital camera was used above the subject's head, and the images are gotten at predefined stop points. However, it is time-consuming in image acquisition and could cause inconsistency problem because the 3D coordinates of the EEG sensors will change if the patient's head moves during the camera rotation. Qian and Sheng [4] propose another single camera photogrammetry system. In their system, both two plane mirrors and a camera are utilized, and the images are acquired simultaneously. However, these two mirrors have been placed with a fixed angle which is about 51.4°. The angle should be neither too big nor too small; otherwise it will affect the images acquisition directly.

Those photogrammetry ways require each EEG sensor mounted on a head to be visible from at least two views. That is to say, two conditions should be satisfied: (i) images captured by the cameras should contain all the sensors; (ii) each sensor should be captured from at least two different views. Those above studies have achieved good results. However, due to those two conditions, all previous studies are based on the use of either a rotating camera or a camera with assisted equipment or multiple cameras (11 or 12). None can realize simultaneous acquisition of images with low cost and a free practical environment.

This paper presents a calibration process for a novel low cost and free practical environment photogrammetry system framework for fast localization of EEG sensors. This method only needs four 3D sensors and does not need special assistant equipment. With the development of Kinect equipment, the 3D information of scene can be accessed easily. But the accuracy of depth obtained by Kinect is not enough for accurately locating EEG sensors. In this paper, in order to achieve accurate results of calibration, an efficient local repairing algorithm has been proposed to inpaint the depth image obtained by Kinect. After repairing the depth image, the accurate location of the EEG sensors can be acquired. Other than the previous photogrammetric studies, the positions of the electrodes are not determined by more than two views. The Kinect can obtain the 3D information of scene. So the 3D location of the electrodes can be achieved from a single view. Compared to obtaining the 3D location of the electrodes by more than two images, it can reduce the amount of calculations. This greatly improves the speed of this system. But these EEG sensors are distributed among four different coordinates. Therefore, a calibration method is proposed which can obtain the conversion relationship among those four perspectives to enhance the practicability of this system.

The remainder of this paper is organized as follows. In Section 2, the repairing algorithm for Kinect depth image is described in detail. In Section 3, a calibration process of this system is proposed. The experimental results are demonstrated in Section 4. Finally, the conclusion of this paper is in Section 5.

## 2. Depth Map Inpainting Algorithm

**Figure 1:**
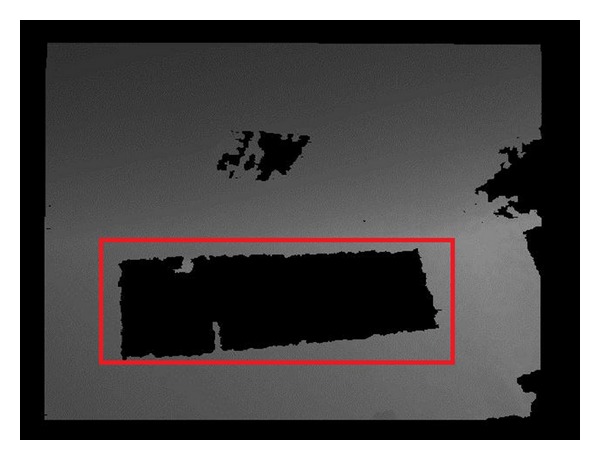
Noise of situation (i).

**Figure 2:**
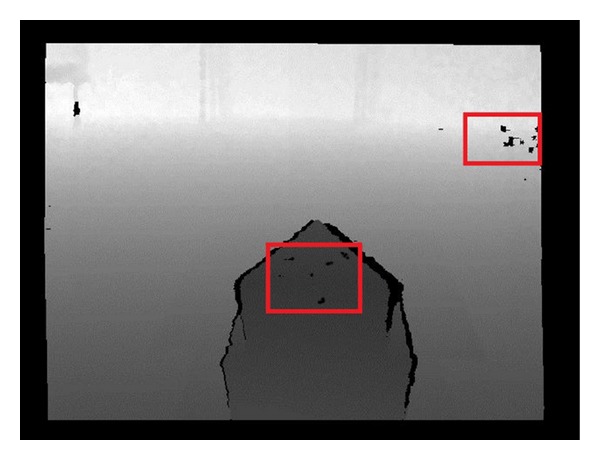
Noise of situation (ii).

Kinect uses an infrared emitter to emit the IR light which people cannot see to get the depth information [5]. A lens placed in front of the IR is used to grate laser light projected on the uniform distribution in the measurement space; then every speckle in the space is recorded through the infrared camera. After getting the origin data, a special chip is used to calculate the depth image. The key of this technology is the laser light speckle. When the laser illuminates the surface of an object, it will randomly form the radioactive spots which are called speckles. Without the reflective speckles in the surface of the object, the depth information of the object cannot be obtained. This is the first kind of depth image noises. And it happens for two reasons generally: (i) special materials, such as glass, mirror, and infrared absorbing materials; (ii) special structures, which cannot reflect speckle. The second kind noise of depth image is the image noise, which is caused by the Kinect software and hardware itself. In general, the deviation of depth image captured by Kinect is about 3 mm when the photographed distance is about 0.8 m~3.8 m. If the deviation is larger than 3 mm, it is considered to have different depth.  Figure 1 shows the first noise of situation (i), and Figure 2 shows the situation (ii).

After knowing the noises, an effective algorithm has been designed to remove those noises. Generally, the image filter can be expressed as

(1) $$\begin{array}{c} I^{\prime }\left(x,y\right)=\frac{\sum_{i,j\in \Omega }w\left(i,j\right)*I\left(i,j\right)}{\sum_{i,j\in \Omega }w\left(i,j\right)}, \end{array}$$

where *I*(*i*, *j*) is an image as in Figures 1 and 2 containing noises, *I*′(*x*, *y*) is the denoised image, and *Ω* is the pixel neighbors of point (*x*, *y*). Generally, *Ω* is the rectangular area with center point (*x*, *y*). *w*(*i*, *j*) denotes the weight of point (*i*, *j*). And *w* is a unique parameter.

After analyzing the noises of depth images captured by Kinect, we use bilateral filter [6, 7] to smooth the depth map firstly. For the bilateral filter, its weight is composed of the spatial weight *w*_*s*_ and weight *w*_*r*_ of gray domain, where *w* is

(2) $$\begin{array}{c} w=w_{s}*w_{r}\\ w_{s}=\exp\left(-\frac{{\left(i-x\right)}^{2}+{\left(j-y\right)}^{2}}{2\sigma_{s}^{2}}\right)\\ w_{r}=\exp\left(-\frac{{||I\left(i,j\right)-I\left(x,y\right)||}^{2}}{2\sigma_{r}^{2}}\right). \end{array}$$

Users decide how close pixel neighbors can be considered for the computation by *σ*_*s*_ and how close should the neighbors' intensity be by *σ*_*r*_ value. Thus, bilateral filter is essentially a modified Gauss filter, using the gray scale information to preserve the edge information while filtering. The weight is adjusted according to the field of gray difference. That is, in the *Ω* domain, if the gray level of point (*i*, *j*) and point (*x*, *y*) is closer, weight *w* is closer to the Gauss weight too. If the *σ*_*s*_ is fixed and *σ*_*r*_ is too large, the weight *w* with difference gray level will be large. Then it losses the effect of preserving edges by the gray change. The bilateral filter changes into the Gauss filter. And if *σ*_*r*_ is too small, the weight *w* is too sensitive on difference gray level difference. Then it will lose the filtering effect.

Therefore, we need to have a general understanding of the noises of the input image. For the depth image obtained by Kinect, the deviation is about 3 mm when the photographed distance is about 0.8 m~3.8 m. So the *w*_*r*_ can be simplified as

(3) $$\begin{array}{c} w_{r}=\{\begin{array}{cc} 1, & \left(\left|I\left(x,y\right)-I\left(i,j\right)\right|\leq 3\right),\\ 0, & \text{else}. \end{array} \end{array}$$

The simplified filtering function greatly reduces the number of computations. Then the depth difference which is greater than 3 mm is judged as a different surface, and its weight is set to 0.

If *I*(*x*, *y*) = 0, then *I*′(*x*, *y*) = 0, as can be calculated by the modified formula. Thus it cannot be fixed by the neighbors which had been known. Considering that the texture information is complete, this paper combines the RGB image with the depth image to fill the missing pixels of depth image [8, 9]. This paper uses the searching algorithm based on SAD matching to find out the best texture matching point *B* in texture image, which relies on the texture of the missing point *A* in the texture image. Then, the pixel value of *A* is replaced by the pixel value of *B*. If the best matching point's depth value is still missing, then the suboptimal point is used to replace the best matching point *B* and so on. In order to improve the processing speed, our algorithm is based on the pixel color matching. And the matching window size is set to 7 to control the search range. Although the matching point is not always the most accurate, the experiment results show that the depth map can be repaired with a good quality.

Fast restoration algorithm based on pixel would result in repaired noises in the repaired area. This paper uses the median filtering with good characteristics to process the depth image after restoration again. Not only does it ensure the speed, but also it maintains the most amount of the original value of the depth image.

## 3. Calibration Method

In previous section, a high reliably depth image was acquired. This is fully prepared for our next work. In this section, a proposed calibration method will be discussed in detail. In this paper, the EEG sensors' 3D positions which are distributed on the patient's head are obtained from four views through the Kinect. Thus, four sets of EEG sensors data are captured. And those four sets of data belong to four different Kinect coordinates. So those four sets of data have to be registered into one set of data. Thus, a calibration process, which is realized to get the conversion relationships among four perspectives simultaneously, has been designed to solve that problem. And this method is running with an efficient calibration board and a calibration body.

### 3.1. The Calibration Board

**Figure 3:**
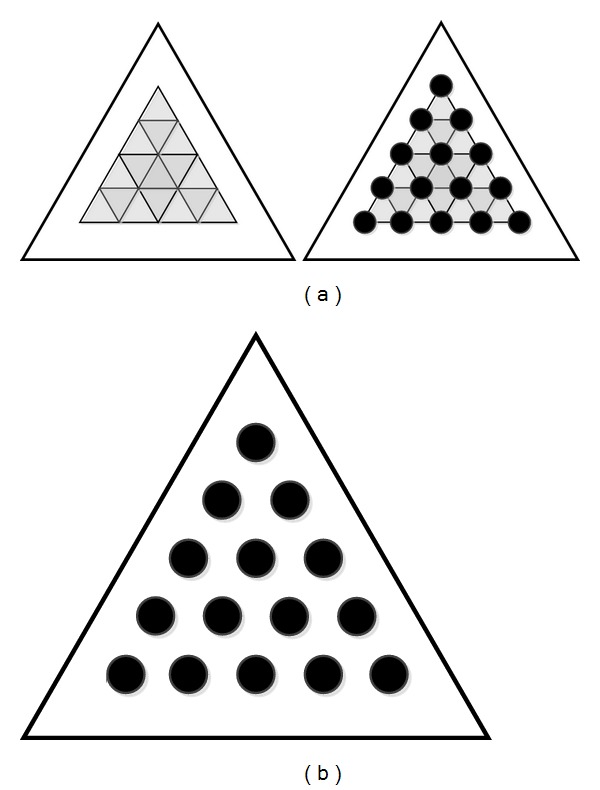
Calibration board which is designed for our calibration process. (a) shows the structure of the calibration board; (b) is the calibration board used in experiment.

As shown in Figure 3, the triangle outside restrains the positions of those solid circles inside. It helps us find all of the solid circles quickly. And these solid circles are arranged according to a certain rule as is shown in Figure 3(a). There is the same distance among adjacent solid circles. And each of the three adjacent centers of the circles constitutes a regular triangle. This design provides us with highly reliable constraints to find the centers of solid circles exactly later. That greatly improves the accuracy of the calibration result. Other than that, choosing the calibration board, as is shown in Figure 3(b), makes us very convenient to have a corresponding public points' pair to calculate the relation of two coordinate systems.

### 3.2. The Calibration Body

**Figure 4:**
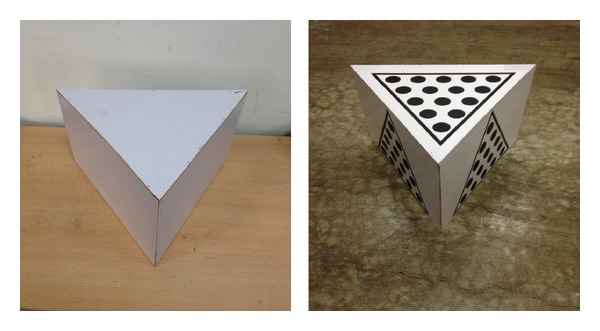
Calibration body helps us to calculate the conversion relationships of four views simultaneously.

The calibration body is a triangular prism structure. The calibration board is put on the top surface and three side surfaces of the triangular prism. And the solid circles on each two surfaces of the calibration body are correspondingly arranged as shown in Figure 4. Each group of centers of solid centers is used to represent one surface. If conversion relationship of each surface of triangular prism is known, each two groups can act as a group of common points to calculate the conversion relationship between the two perspectives of the two surfaces. So this calibration process is divided into two parts.

### 3.3. The Calibration Process

**Figure 5:**
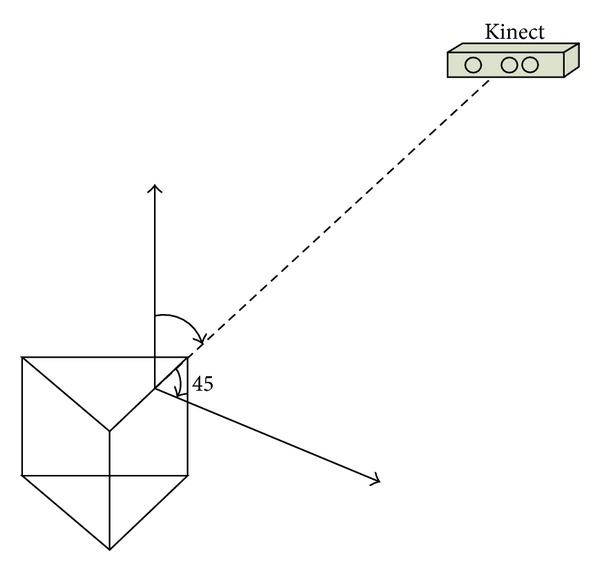
Kinect gets data by facing the line of intersection of the top surface and the side surfaces of the calibration body.

**Figure 6:**
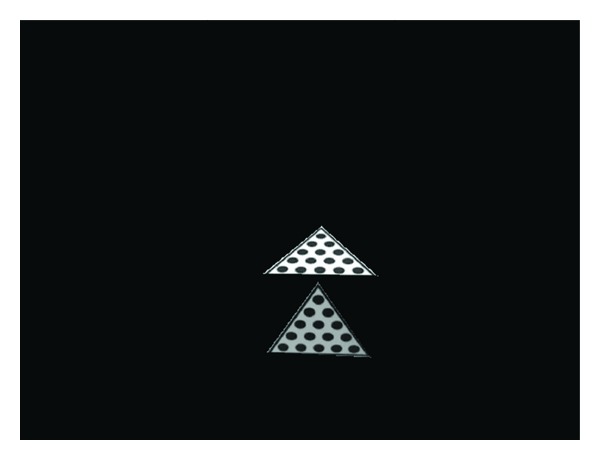
Identifying the solid circles of the top surface and solid circles of the side surfaces through triangle.

The first part is self-calibration of the triangular prism itself. The conversion relationships between the top surface and three side surfaces of the triangular prism will be figured out. Kinect gets data by facing the line of intersection of the top surface and the side surface as in Figure 5. The triangle on the calibration board not only allows us to find all the solid circles quickly, but also helps us to identify the solid circles of the top surface and solid circles of the side surfaces convenient as shown in Figure 6.

Then, the locations of all solid circles are captured through connected component labeling [10–13]. The centroid of each region has been used to represent each solid circle. Thus, there is a set of data *T* representing the top surface and other three sets of data, S1, S2, and S3, representing the side surfaces. The conversion relationships among the top surface and side surfaces are calculated by *T* and S1, S2, and S3. From the three-dimensional coordinate conversion process, the conversion model can be obtained as follows:

(4) $$\begin{array}{c} {\left(\begin{array}{c} X\\ Y\\ Z \end{array}\right)}_{T}=\left(\begin{array}{c} \Delta X\\ \Delta Y\\ \Delta Z \end{array}\right)+\left(1+m\right)R{\left(\begin{array}{c} X\\ Y\\ Z \end{array}\right)}_{S}, \end{array}$$

where (*X* *Y* *Z*)_*S*_^*T*^ is the coordinates in the original coordinates, (*X* *Y* *Z*)_*T*_^*T*^ denotes the coordinates of the target coordinates, (Δ*X* Δ*Y* Δ*Z*)^*T*^ is a transformation factor, and *m* is the scale parameter; generally, *k* = 1 + *m*, and *R* is the rotation matrix, instead of classical rotating angle form to improve the computing speed [13–16]. The Rodrigues matrix has been used to represent the rotation matrix to solve this model.

**Figure 7:**
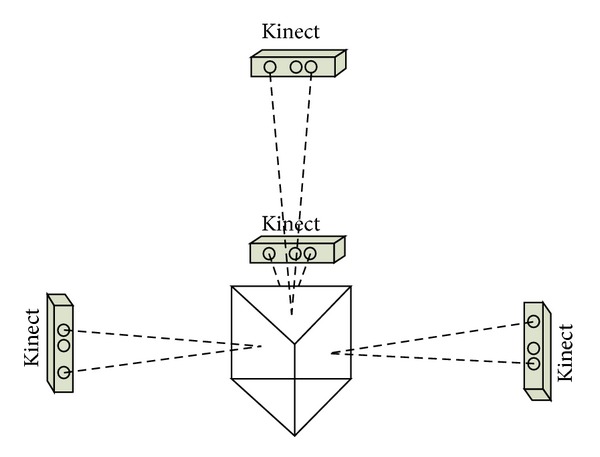
Four Kinects capture data from four views by facing the top surface and side surfaces of the calibration body.

In the second part, four Kinects are used to get data as shown in Figure 7. The conversion relationships between the top surface and sides of the triangular prism have been calculated out in the first step. Thus, the centers of the solid circles on the side surface and the centers of the solid circles on the top surface can be used to calculate the conversion relationship of those two views through solving the model described above. Thus, the conversion relationships among those four views have been calculated out simultaneously.

## 4. Experimental Results

**Figure 8:**
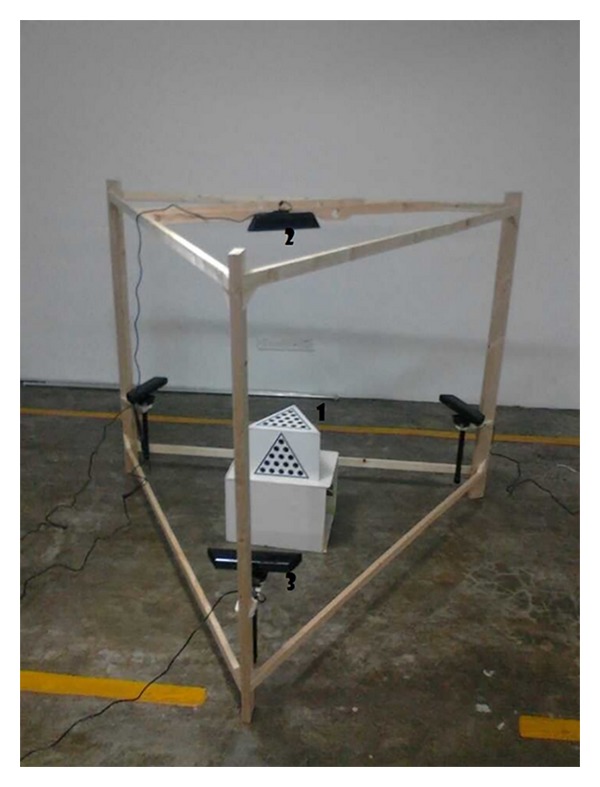
Experimental environment, image data acquisition system used in this study. The object is a triangular prism calibration body (1) designed with a designed calibration board. (2) and (3) are Kinect equipment to obtain the images. Four Kinects as shown in Figure 8 obtain images in four different views by facing a surface of triangular prism.

The experimental apparatus is shown in Figure 8. It is part of the experiment of fast determining EEG sensors positions. This paper presents a local repair algorithm for depth images captured by Kinect and a fast accuracy method of calculating the conversion relationships of four views simultaneously. Experiments demonstrate that accuracy calibration results can be achieved quickly. Thus it can be used in our novel photogrammetry system for fast determining EEG sensors positions on a head. This paper performs the experiments with computer configurations of 2.00 GHZ (CPU), 2.00 G (RAM). The experimental images in this paper are obtained by Kinect equipment.

### 4.1. Experiments with Depth Image Inpainting

**Figure 9:**
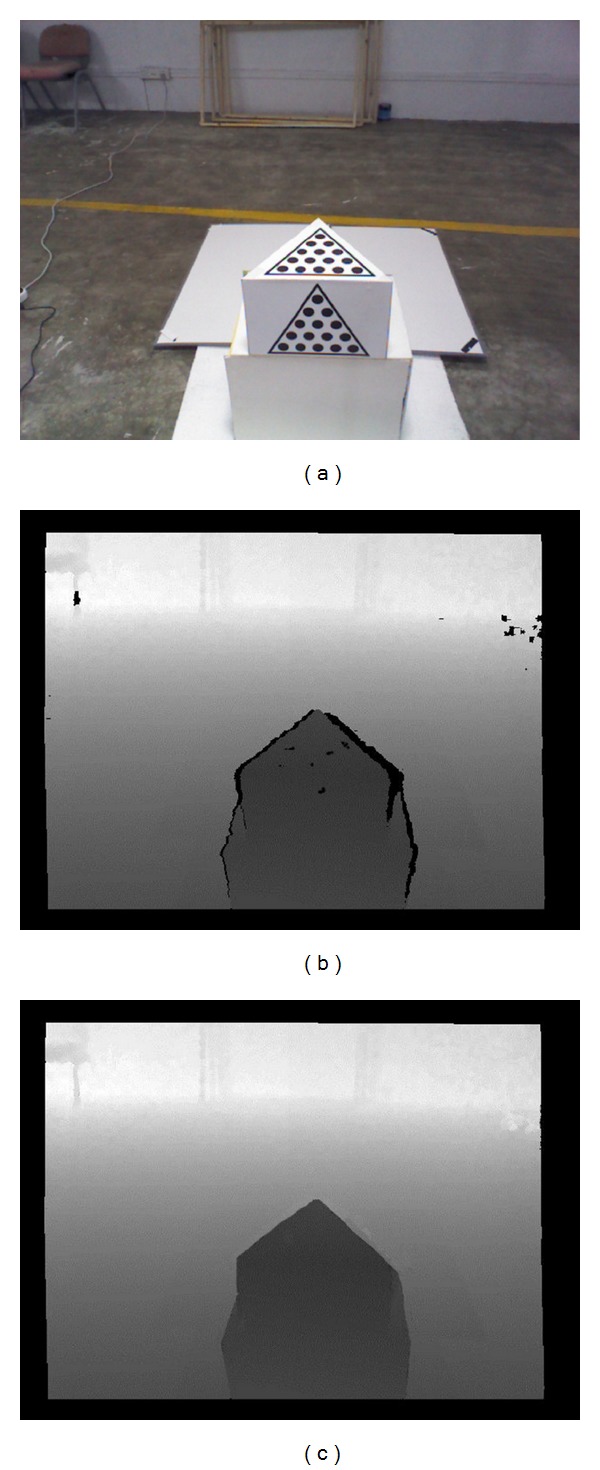
Comparison of the depth image. (a) RGB image, (b) depth image without inpainting, and (c) depth image after inpainting.

The improved bilateral filter and the SAD matching algorithm are applied to inpaint the depth image captured by Kinect. Objective comparison of the experimental results is given. The comparison of the depth image is shown in Figure 9. It shows that a good repaired image has been achieved, and it shows that this local repair algorithm is competitive.

### 4.2. Experiment with Calibration Process

Firstly, self-calibration of the triangular prism is presented. The conversion relationships between the top surface and three side surfaces of the triangular prism will be figured out as is shown in Figure 5. Then we use the calibration body to calculate the conversion relationship among those four views out.

After calculating the conversion relationships of those four views, this paper uses corners of a 6∗8 chessboard plate to act as common points to verify our calibration results. The absolute conversion error of the proposed calibration process is calculated as given in

(5) Δd=ΔX2+ΔY2+ΔZ2=(X−X′)2+(Y−Y′)2+(Z−Z′)2,

where *X*, *Y*, and *Z* are coordinates in destination view and *X*′, *Y*′, and *Z*′ are transformed coordinates from one view to the destination view.

Analysis of errors is shown in Table 1. The average error is 1.07 mm. Previous studies about 3D coordinates transformation show that the accuracy of result of our results is competitive. And as we can see from studies about photogrammetry system, this calibration process has a good accuracy. It is suggested that this calibration process can be used in our novel photogrammetry framework for EEG source localization applications in the human brain, for example, for analysis of neural activities [17, 18].

## 5. Conclusion

The paper presents a calibration process for a novel photogrammetry framework for fast localizing the EEG sensors. More specifically, we designed a local repair algorithm for depth images captured by Kinect and a special calibration process for calculating the conversion relationships of four views simultaneously for this framework. Experiment results have demonstrated that this novel photogrammetry framework is practical according to the speed of this system and errors of this calibration process.

## Figures and Tables

**Table 1:** Errors of 48 corners of 6 ∗ 8 chessboard plate transform from one coordinate to another by using the calibration results. Unit of those errors is mm.

	Δ*X* (mm)	Δ*Y* (mm)	Δ*Z* (mm)	Δ*d* (mm)
1	−0.04	−0.90	−0.11	0.91
2	0.91	−0.79	−0.67	1.38
3	−0.21	−0.79	−0.14	0.83
4	0.57	0.08	−0.45	0.73
5	0.95	0.33	−0.87	1.33
6	0.70	0.28	−0.43	0.87
7	−0.86	−0.35	−0.92	1.31
8	0.45	−0.12	−0.85	0.97
9	−0.58	−0.74	−0.40	1.02
10	0.22	0.95	−0.47	1.08
11	0.50	0.52	0.24	0.76
12	0.50	0.30	−1.42	1.54
13	−0.06	−0.68	−0.84	1.08
14	−0.02	−0.43	−0.98	1.07
15	−0.68	−0.89	−0.47	1.21
16	0.50	−0.41	−0.03	0.65
17	0.98	0.33	−1.00	1.44
18	0.37	0.73	−0.41	0.92
19	−0.07	−0.56	−0.88	1.05
20	−0.97	−0.09	−0.66	1.18
21	−0.88	−0.18	−0.65	1.11
22	1.04	0.29	−1.15	1.58
23	0.04	0.35	0.68	0.77
24	0.57	0.86	0.87	1.35
25	−0.22	−0.18	0.37	0.47
26	−0.94	−0.98	−0.15	1.37
27	−0.04	0.83	0.45	0.94
28	0.69	0.77	0.93	1.39
29	0.30	0.71	0.60	0.98
30	0.88	0.66	0.36	1.16
31	−0.06	−0.18	0.59	0.62
32	−0.64	−0.27	0.38	0.79
33	−0.96	−0.63	0.45	1.23
34	−0.45	0.70	0.86	1.20
35	0.74	0.44	1.29	1.55
36	0.22	0.76	0.73	1.08
37	−0.71	−0.61	0.28	0.98
38	−0.18	−0.09	0.27	0.34
39	0.21	0.29	0.81	0.89
40	−0.56	0.97	0.11	1.13
41	0.16	0.41	0.25	0.51
42	1.59	0.09	0.26	1.61
43	−0.01	−0.42	0.90	0.99
44	−0.10	−1.75	0.53	1.83
45	−0.63	−0.05	0.90	1.10
46	−0.36	0.47	0.47	0.76
47	1.24	0.21	0.69	1.43
48	−0.11	0.74	0.68	1.01
